# Platform technologies and human cell lines for the production of therapeutic exosomes

**DOI:** 10.20517/evcna.2020.01

**Published:** 2021-03-30

**Authors:** Jiyoon Kim, Yonghee Song, Cheol Hyoung Park, Chulhee Choi

**Affiliations:** ^1^ILIAS Biologics Inc., Daejeon 34014, South Korea.; ^2^Department of Bio and Brain Engineering, KAIST, Daejeon 34141, South Korea.; ^#^Authors contributed equally.

**Keywords:** Therapeutic exosome, exosome engineering technologies, human cells, stem cells, HEK293, dendritic cells

## Abstract

Exosomes are extracellular vesicles secreted by most cell types and represent various biological properties depending on their producing cells. They are also known to be important mediators of intercellular communication. Recent data suggest that exosomes can mediate the therapeutic effects of their parental cells; hence, they have been in the spotlight as novel therapeutics. To develop and manufacture effective therapeutic exosomes, customized strategies are needed to use appropriate technologies for exosome engineering and to select suitable production cell lines. In this review, we provide an overview of currently available exosome engineering platform technologies for loading active pharmaceutical ingredient cargo and the types of human cells/cell lines that are being used as exosome-producing cells, particularly focusing on their characteristics, advantages, and disadvantages.

## INTRODUCTION

Extracellular vesicles (EVs) are nanometer-sized membrane-encapsulated vesicles which are secreted by most types of cells. They can mediate cell-to-cell communication through their bioactive cargo molecules, such as nucleotides, lipids, and proteins. The three major subtypes of EVs are exosomes, microvesicles, and apoptotic bodies^[1,2]^. These EV subtypes can be distinguished based on their size and mode of biogenesis. Microvesicles and apoptotic bodies have a diameter of 0.05-1 m and 1-5 m, respectively, whereas exosomes are relatively smaller with a typical diameter of 30-200 nm^[3]^. These small secretory particles, “exosomes”, are released from most eukaryotic and prokaryotic cells into the extracellular fluids, such as blood, plasma, urine, saliva, milk, semen, and tears. In recent years, of the three EV subtypes, exosomes have attracted attention as a therapeutic drug (e.g., NCT04173650) or vehicle for drug delivery based on several groundbreaking studies conducted on their therapeutic potential^[4]^.

**Figure 1:**
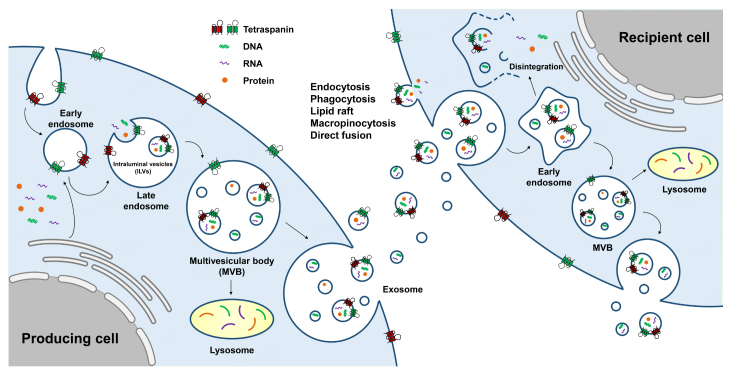
Schema describing biogenesis of exosomes. Biogenesis of exosomes starts with the first invagination of plasma membrane of the producing cells, followed by the formation of early endosomes. Then, the cytosolic components (nucleic acids and proteins) are loaded into ILVs of the late endosomes through the second invagination. After the maturation of MVBs, some components undergo degradation by fusion with lysosomes, while others are secreted into the extracellular fluids as “exosome”. Exosomes enter the recipient cells via various mechanisms, such as endocytosis, phagocytosis, lipid raft, macropinocytosis, and direct fusion. After being taken up by the recipient cells, exosomes fuse with early endosomes and release their cargos into the intracellular area by disintegration. Other parts of the exosomes either are released back to the extracellular fluid by fusion with transmembrane or get degraded by lysosomes. ILVs: intraluminal vesicles; MVBs: multivesicular bodies.

Exosomes have a different biogenesis pathway compared to that of other EVs. Exosome biogenesis is a multi-step process which starts with invagination of early endosomes from plasma membrane of the producer cells [Figure 1]. As previously reported, early endosomes mature into multivesicular bodies (MVBs) through the late endosomal stages. In the middle of MVB maturation, a second invagination called “membrane inward budding” of the exosome generates intraluminal vesicles (ILVs) inside the late endosomes. When ILVs bud inward of late endosomes, various cytoplasmic bioactive components, including proteins, lipids, and nucleic acids, are loaded into ILVs as payloads. In a few cases, early endosomes are produced directly by the endoplasmic reticulum or trans-Golgi, independent of membrane invagination^[5,6]^. MVBs are fused with lysosomes for degradation or with plasma membrane of the parent cells to release multi-components, including ILVs, into the extracellular fluids. The released materials are referred to as exosomes^[7-9]^.

Much evidence exists for exosome biogenesis; however, understanding of their cellular uptake is in its infancy. To track the functionality, tropism to tissue/organ, and *in vivo* stability of exosomes, their cellular uptake mechanisms should be proposed. The internalization of exosomes into the cells involves distinct mechanisms, such as endocytosis^[10-12]^, phagocytosis^[11]^, micropinocytosis^[13]^, and direct fusion^[14] ^[Figure 1]. However, it is still unclear how the recipient cells respond differently for each exosome uptake mechanism. After being taken up by the recipient cells, exosomes enter the early endosomes and undergo one of the three fates: disintegrate to release internal components, fuse with lysosomes for degradation, or are released back into the extracellular fluid^[9]^.

Many studies are ongoing to utilize the properties and biogenesis of exosomes in developing therapeutics for various intractable diseases. Specifically, various engineering technologies have been developed to load therapeutic biomolecules into exosomes for efficient delivery. To improve the efficacy and productivity of therapeutic exosomes, it is very important to identify the optimal exosome-producing cells, and various cells are being examined at academic institutions and pharmaceutical companies. In this review, we discuss current platform technologies for developing therapeutic exosomes and the exosome-producing human cells/cell lines.

## PLATFORM TECHNOLOGIES FOR THERAPEUTIC EXOSOMES

**Figure 2:**
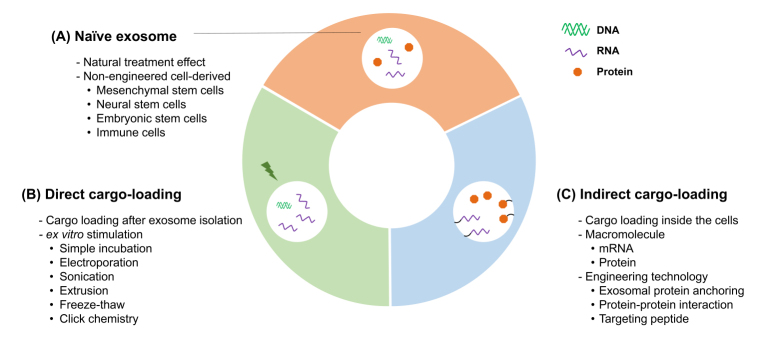
Platform technologies for therapeutic exosomes can be categorized into three subtypes. (A) Naïve exosomes are isolated from different types of cultured cells including stem cells and immune cells. The major limitation of this method is the control over type and amount of cargo molecules. (B) The direct cargo-loading method uses ex vitro stimulations after exosome isolation. Limitations with this method include exosome stability, productivity, and the size of the cargo molecules. (C) In the indirect cargo-loading method, selected and specific macromolecules can be loaded into exosomes by engineering producer cells. Currently, this technique is not only used for loading therapeutic macromolecules but also for targeting specific tissues and organs.

Exosomes have various biological characteristics, such as biocompatibility, high penetrability, and biodegradability^[15]^. Although a lot of supportive evidence for exosome therapeutics is still needed, the unique biological properties of exosomes make it possible to expect therapeutic potential in the clinic. Exosomes are composed of various surface and intracellular molecules, reflecting the type and condition of their parental cells. They are found in all kinds of body fluids, making them suitable candidates for liquid biopsy. Exosomes not only can be used as diagnostic markers, but also can act as drug delivery vehicles or exert therapeutic effect itself, because of their characteristics to elicit minimal host immune responses^[16,17]^ and ability to cross the biological barriers in the brain or placenta^[18-20]^. As reported, repeatedly injecting engineered exosomes specifically delivered RNAi to the mammalian host brain, while inducing minimal immune response^[19]^. Because of the advantages of using exosomes as a drug delivery vehicle, the research for pharmaceutical exosomes has advanced significantly in recent years^[21]^. There are three different approaches to using exosomes as therapeutics [Figure 2].

### Naïve exosome

The first is to use naive exosomes that are isolated from various cell types, such as mesenchymal stem cells (MSC), embryonic stem cells, and immune cells, directly for disease treatment as is^[22,23]^. Although naïve exosomes are regarded as promising therapeutic agents, their exact pharmacological mechanisms are poorly understood. Hence, there is an imperative need to better understand the complexity of naturally secreted exosomes and to identify the components with therapeutic activities.

### Direct cargo-loading

There are attempts to develop therapeutic exosomes by loading active pharmaceutical ingredients into exosomes using direct or indirect loading. The direct loading methods deliver therapeutic molecules to inside of the exosomes through various incubation methods, such as simple incubation or physical, chemical, or electrical stimulation. As an example of simple incubation, when EL-4-derived exosomes and curcumin were co-cultured at 22 °C, the exosome-encapsulated curcumin exhibited enhanced anti-inflammatory effects on sepsis mouse model compared to curcumin alone^[24]^. Similarly, when chemotherapeutic agent paclitaxel (PTX) was incubated with exosomes by shaking at 37 °C, it was incorporated into exosomes, and these engineered exosomes showed at least 50-fold enhanced cytotoxic activity compared to PTX alone^[25]^. Furthermore, electroporation and sonication are generally used for loading nucleotides^[19]^. For instance, exosomes loaded with RNAi and small-RNAs in the lumen reduced the effects of oncogenes in tumor cells^[26,27]^. Other cargo loading methods include extrusion, freeze-thaw, and click chemistry^[28,29]^. Direct cargo-loading allows a more tailored therapeutic effect of exosomes by loading the desired molecule compared to naïve exosomes. However, the major impediment of these techniques is their low stability^[30-32]^ and unsuitability for high molecular-weight cargos, such as mRNAs and proteins.

### Indirect cargo-loading

Several pioneering groups have reported a new generation of techniques with indirect cargo loading. In recent years, diverse loading methods have been introduced without disrupting the structure and function of large size RNA molecules^[33,34]^. Fundamentally, these techniques use RNA binding proteins, which are known to interact with specific sequences of RNA molecules. In 2018, Kojima *et al.*^[33]^ introduced a set of devices known as EXOsomal transfer into cells (EXOtic) and demonstrated its applicability using the interaction between C/D box RNA structure and L7Ae ribosomal protein. In detail, C/D box RNA was subsequently conjugated after therapeutic mRNA synthesis, and L7Ae was fused with CD63, which is naturally localized on the exosomal membrane. With this, mRNA was successfully loaded into exosomes^[33]^. In the same year, Wang *et al.*^[34]^ developed a method to load mRNA into exosomes based on the interaction between trans-activating response (TAR) sequence and trans-activator of transcription (Tat) protein. They conjugated mRNA with TAR element and membrane protein ARMMs with Tat.

In addition to large size nucleotide loading, protein loading methods have been developed. The direct fusion of exosomal transmembrane proteins including tetraspanins (CD9, CD63, and CD81)^[1]^ with cargo proteins has the potential to load specific cargo proteins into the exosomes. However, the functional area of the cargo proteins from direct fusion is limited only to the close proximity of the recipient cell’s membrane. A platform that addresses this limitation is the “exosomes for protein loading via optically reversible protein-protein interaction” (EXPLOR^®^), which delivers cargo proteins into the cytosol in a free form^[35]^. Cargo protein and tetraspanin, especially CD9, interact with each other through the fusion with cryptochrome 2 (CRY2) and the truncated domain of calcium- and integrin-binding protein 1 (CIBN), respectively. CRY2 and CIBN bind with each other when exposed to specific wavelengths of blue light, and they dissociate in the absence of blue light. This selective interaction allows cargo proteins to be freely localized in the recipient cells. In addition, methods for targeting the therapeutic molecules to specific tissues have been devised.

Similar to cargo loading, membrane proteins are used as an anchor of targeting peptides. For example, Tian *et al.*^[36] ^fused the exosomal membrane protein Lamp2b with the αv integrin-specific RGD (R, arginine; G, glycine; D, aspartic acid) peptide, which specifically targeted αv integrin expressing tumor cells and reduced the tumor progression. Most of the cargo loading approaches introduced above require not only engineering exosome-producing cells but also ways to increase exosome production. Therefore, it is important to select cells that are amenable for transfection and large-scale culture processes.

## EXOSOME PRODUCING HUMAN CELL LINES SUITABLE FOR THERAPEUTIC APPLICATIONS AND SCALE-UP PRODUCTION

While exosomes have conserved proteins such as tetraspanins (CD81, CD63, and CD9), Tsg101, and Alix, which are responsible for their common biological activities, they also have specific components depending on their producer cell types, suggesting that exosomes can reflect the intrinsic properties of the producer cells. For example, miR-146a, which contributes to the protection against myocardial infarction, is highly enriched in the exosomes released from cardiosphere-derived cells (CDCs)^[37]^. Similarly, a high concentration of anti-inflammatory miR-223 is found in exosomes released from human peripheral blood mononuclear cells (PBMCs) and animal bone marrow-derived mesenchymal stem cells (MSCs)^[38-40]^. As the therapeutic effects of exosomes can be modulated not only by the type of parental cells but also by the environmental factors such as culture materials and conditions, the characteristics of cells should be carefully considered to select the suitable exosome-producing cell lines for basic research as well as for clinical applications. Scalability, consistency, and controllable manufacturing methods for culture will need to be established to produce clinical-grade exosomes. In the following section, we compare the genetic modification, productivity, scalability, and safety of exosome producing human cell lines and summarize research trends of several human cells currently in use for production [Table 1].

**Table 1:** Advantages and disadvantages of cells/cell lines used for production of therapeutic exosomes in preclinical studies

**Cells**	**Advantages**	**Disadvantages**	**Exosome type**	**Target diseases**
MSCs	Immunomodulatory; low immunogenicity	Limited scalability (adherent); limited growth capacity	Naïve	Myocardial ischaemia / reperfusion injury^[41-43]^ Traumatic brain injury^[44,45]^ Alzheimer’s disease^[46,47]^ Lung injury^[48]^ Graft-versus-host-disease (GvHD)^[49]^ Cutaneous injury (wound)^[50]^ Kidney injury^[51]^
			Engineered	Tumor^[52,53]^ Stroke^[54]^ Rheumatoid arthritis^[55]^ Acute lung injury^[56]^ Myocardial infarction^[57]^
NSCs	Immunomodulatory; low immunogenicity	Limited scalability (adherent)	Naïve	Spinal cord injury^[58]^ (Ischemic) stroke^[59]^ Alzheimer's disease^[60]^ Hypoxia-reperfusion injury^[61]^
			Semi-engineered (culture media-modified)	Spinal cord injury^[62]^
HEK293	High scalability (possible to suspension); High growth capacity (immortalized cell line); Easy manipulation	Tumor metastatic potential	Engineered	Drug addiction^[63]^ Tumor^[64-67]^ Parkinson’s disease^[33]^ Sepsis^[68]^ Heart disease^[69]^ Alzheimer's disease^[70]^
DCs	Immunomodulatory; low immunogenicity	Limited scalability (adherent)	Engineered	Alzheimer's disease^[19]^ Parkinson’s disease^[71]^ Cancer (vaccine)^[72,73]^
CDCs	Low immunogenicity	Limited scalability (adherent)	Naïve	Myocardial infarctio^[37,74]^ Duchenne muscular dystrophy^[75]^
Amniotic cells	Low immunogenicity	Limited scalability (adherent)	Naïve	Ovarian dysfunction^[76]^ Wound healing^[77,78]^ Lung injury^[79,80]^
CAR-T	Low toxicity; low immunosuppression	Limited scalability	T cell-engineered	Tumor^[81]^

MSCs: Mesenchymal Stem Cells; NSCs: neural Stem Cells; HEK293: human embryonic kidney 293 cells; DCs: dendritic cells; CDCs: cardiosphere-derived cell; CAR-T: chimeric antigen receptor-T cells.

### Stem cells: mesenchymal stem cells and neural stem cells

MSCs are multipotent stem cells that can be isolated from various human tissues including bone marrow, adipose tissue, amniotic fluid, umbilical cord blood, and others. They can be differentiated into multiple lineages such as adipocytes, chondrocytes, and osteocytes. These cells exhibit therapeutic effects and have been broadly used in clinical trials^[82,83]^. Especially, MSCs have been in the spotlight as source cells for regenerative medicine for various diseases including myocardial ischemia / reperfusion injury^[84]^, graft-versus-host-disease (GvHD)^[85]^, Alzheimer’s disease^[86]^, and skin injury^[87]^. Recently, it has been reported that MSC-derived exosomes have similar therapeutic effects to those of MSCs, suggesting that MSC-derived exosomes can also be used as therapeutic agents^[88]^. Similarly, the therapeutic effects of neural stem cells (NSCs) such as regeneration, plasticity, neurogenesis, and attenuated neuroinflammation in neurodegenerative disease have been reported to be mediated by NSC-derived exosomes which are enriched with specific miRNAs^[89]^.

The MSC- and NSC-derived exosomes are known to have therapeutic potentials on their own without going through any engineering. Therefore, stem cells such as MSCs and NSCs that possess natural intrinsic therapeutic effects could be a good source for producing naïve exosomes^[89,90]^. To produce MSC-derived naïve exosomes, either autologous or allogeneic cell sources can be used depending on their advantages and limitations. While autologous cells are non-immunogenic, they have limitations with scalability. In contrast, allogeneic cells have the opposite characteristics.

#### Productivity of exosomes

In terms of exosome productivity, MSCs produce a relatively large number of exosomes per cell^[82,91]^. However, for more efficient and scalable production of therapeutic exosomes, requirements such as use of chemically defined materials and serum-free suspension culture should be met. The current conventional *in vitro *monolayer culture method used for MSCs is a major limitation to scale up the production of therapeutic exosomes. Recently, a three-dimensional culture method was reported to increase the productivity of MSC-derived therapeutic exosomes^[92]^, although the growth capacity of the cells was limited. Immortalization of MSCs by introducing oncogenes such as c-myc or human telomerase reverse transcriptase is being considered to provide MSCs with sustained growth capabilities^[91,93]^. Furthermore, the conditionally immortalized hNSC line, generated by c-MycER^TAM^ technology^[94]^, fulfilled the needs of brain research field and clinical market by enabling large-scale cell production, and hNSCs are being studied in clinical trials for stroke and critical limb ischemia conditions^[95]^. This suggests that the exosomes produced by immortalized hNSC cells may also have therapeutic effect. However, the immortalization strategy requires careful attention, as it poses potential safety issues such as tumorigenicity^[96]^.

#### Safety

MSC-derived exosomes have advantages with regards to safety. MSCs are considered to be non-immunogenic with a lower risk of allogeneic immune rejection by the host^[90]^, and they are currently undergoing extensive clinical trials. However, the pro-proliferative effects of MSC-derived exosomes on injured cells suggest the possibility of their involvement in cancer progression, even though the pro- or anti-cancer effect of MSC-derived exosomes is currently controversial^[97-99]^. Another safety concern with stem cells is the use of animal-derived serum for cell growth. If serums such as fetal bovine serum (FBS) are not completely removed before the final exosome production, there is a high possibility that these serum contaminants will be loaded into the exosomes during the culture. The retention of these serum contaminants in exosomes can be a problem from a regulatory standpoint in the production of therapeutic agents. Alternatively, using xeno-free culture media components or exosome-depleted FBS could be considered; however, this may affect the compositions or physiological properties of exosomes and result in stress-induced phenotype by loading reactive oxygen species and stress-related proteins^[100]^.

### HEK293 cells

HEK293 is a human embryonic kidney cell line and has been commonly used in research and bio-industrial field for more than 30 years^[101]^. HEK293 cells have various technological advantages required for the development of biopharmaceuticals such as rapid growth, simple culturing, and easy manipulation, owing to their high transfection efficiency. In addition, HEK293 has been widely used as a cellular model in research for producing exosomes as drug carriers due to its high exosome productivity and neutral phenotype^[68,102]^.

#### Productivity of exosomes

For high scalability of biopharmaceutical productions including therapeutic exosomes, it is advantageous for the producing cell lines to be adapted to single-cell suspension culture at high density. HEK293F cell line, developed by Invitrogen^[103]^, has been adapted to grow in serum-free suspension culture and is useful for the large-scale production of industrial bio-products. Several biopharmaceuticals produced using HEK293 cells have been recently approved by the US Food and Drug Association (FDA) or European Medicines Agency (EMA), suggesting that HEK293 cells can be utilized for clinical-grade biological production during the development of new therapeutics^[104]^.

#### Engineering of exosomes

To produce engineered exosomes loaded with specific regulatory genes or proteins with enhanced targeting ability, efficient transfection protocols are needed. HEK293 cell line is extensively used in exosome production because of its ease of handling and transfection. For instance, HEK293T cells engineered with “EXOtic” devices were designed to load therapeutic mRNAs in exosomes to deliver cargoes to specific targets, and they have been used for the production of therapeutic research exosomes^[33]^. In addition, a HEK293T cell line engineered to stably express specific recombinant proteins using EXPLOR^®^ technology allowed loading target proteins into exosomes^[68]^.

#### Safety

Although HEK293 is not a cancer cell line, there may be concerns regarding the tumorigenic or toxic potential of the exosomes derived from these cells because the cell line is immortalized. However, omics research on HEK293T-derived exosomes indicated the enrichment of only a few disease- or cancer-related components and demonstrated their insignificant relationship with the physiological and pathological processes, suggesting that HEK293T-derived exosomes are safe to be used as *in vivo* drug delivery vehicle^[105]^. Furthermore, treatment with varying doses of HEK293-derived exosomes neither had a significant effect on viability and function of other cells such as macrophages and HepG2 nor induced pro-inflammatory cytokine response^[106,107]^. In addition, administration of mice with HEK293-derived naïve and engineered exosomes for several weeks did not induce any significant toxicity^[17]^. These results demonstrate that HEK293-derived exosomes have low *in vitro* and *in vivo* toxicity and immunogenicity.

### Dendritic cells

Dendritic cells (DCs) are antigen-presenting cells that induce an antigen-specific T-cell immune response and mediate between innate and adaptive immunity. The unique characteristic of DC-derived exosomes (Dex) is their ability to present antigen and immune-related proteins (major histocompatibility complex I and II); hence, they can induce an anti-tumor immune response followed by tumor regression. Based on these attributes, Dex has been used for cancer vaccination and treatment in several preclinical and clinical studies^[72,108,109]^. Three phase I clinical trials and one phase II clinical trial with Dex have been completed or are ongoing^[109]^. In one of the phase I clinical trials, Dex was produced using autologous monocyte-derived DCs loaded with antigenic HLA-presented peptides of melanoma-associated antigen from a single leukapheresis for cancer vaccine generation^[110]^.

#### Engineering and productivity of exosomes

Dex can be engineered by modifying the producer cells through transfection^[111]^ or viral transduction^[112]^. For instance, to express neuron-specific rabies viral glycoprotein (RVG) peptides on the surface of Dex and target them to neurons and microglia in the brain, DCs were genetically modified by transfection with plasmid DNA encoding a fusion protein composed of an exosomal membrane protein, Lamp2b, and RVG peptides^[19]^. Furthermore, DCs have been modified by viral transduction to produce tumor antigen-presenting exosomes^[73]^. Since immature DCs have an inherent ability to take up proteins and peptides from surrounding fluids and tissues, special loading techniques are not required to load proteins and peptides into Dex^[109]^. Therefore, model tumor antigens such as ovalbumin (OVA) were co-incubated with immature DCs to obtain OVA-loaded exosomes^[113]^. However, immature DCs produce limited numbers of exosomes^[19]^.

#### Safety

Dex have been proven to be safe in clinical trials^[114]^. Furthermore, exosomes produced from self-derived immature DCs are devoid of immunostimulatory surface markers such as CD40, CD86, and MHC-II, which reduces their immunogenicity^[115]^. Two of the phase I clinical trials demonstrated low toxicity of exosomes derived from tumor peptide-loaded DCs in metastatic melanoma or non-small cell lung cancer patients^[116]^. However, in a recent phase II clinical trial in non-small cell lung cancer (NSCLC), exosomes derived from DCs matured by interferon-gamma and loaded with MHC class I- and class II-restricted cancer antigens did not meet the primary endpoint of 50% non‐progressors by post‐chemotherapy^[117]^. Furthermore, mature DC-derived exosomes have been reported to increase endothelial inflammation and atherosclerosis^[118]^; hence, the use of Dex as a therapeutic agent requires in-depth research and additional controls.

### Others: CDCs, amniotic cells, and CAR-T cells

CDCs improve angiogenesis and restore heart function after myocardial infarction by secreting exosomes^[74]^. The microRNA miR-146a is highly enriched in the CDC-derived exosomes and plays an important role in thickening up of the infarction walls^[37]^. Recent studies have reported that CDC-derived exosomes might be potentially useful in treating Duchenne muscular dystrophy by restoring skeletal muscle and heart functions^[75]^.

CDCs were immortalized using simian virus 40 large and small T antigens. The use of imCDC^sh-test^-derived exosomes as therapeutics is currently being investigated in preclinical research for cell-free therapies^[119]^.

CDCs express MHC I but not MHC II; hence, they evade recognition by CD4 lymphocytes and have low immunogenicity. Xenogeneic CDC-derived exosomes are primarily safe as they induce low immunogenicity^[74]^.

Human amniotic epithelial cells (hAECs), found mainly in the placenta, preserve the properties of embryonic stem cells such as anti-inflammatory and low immunogenicity, making them suitable for regenerative medicine. Recent studies have reported that hAEC-derived exosomes enhance lung repair after injury^[79]^, expedite wound healing^[77]^, and restore ovarian function^[76]^. Furthermore, there are no ethical issues in using amniotic cells and its exosomes. Owing to these advantages, amniotic cells can be considered as one of the suitable candidates for therapeutic exosome research.

Chimeric antigen receptor (CAR)-T cells are emerging as novel immunotherapy agents for a wide range of cancers, and numerous clinical trials have been conducted on CAR-T cells. Recently, two CAR-T cell therapies, Kymriah from Novartis and Yescarta from Kite Pharma, were approved by the US FDA for treatment of diffuse large B-cell lymphoma and B-cell acute lymphoblastic leukemia^[120]^. Similar to other exosomes, CAR-T cell-derived exosomes express CAR-T cell-specific factors, such as CAR and cytotoxic molecules on their surface, and have potential as excellent cancer-targeting and anti-cancer agents^[81]^. Unlike CAR-T cells, the exosomes derived from CAR-T cells do not cause toxicity. Moreover, they do not express programmed cell death protein 1 (PD-1) on their surface, so their anti-cancer effects do not weaken. This suggests that CAR-T exosomes could be used as anti-cancer immunotherapy agents in the future^[81]^.

## THERAPEUTIC APPROACHES OF EXOSOMES

The therapeutic effect of exosomes derived from different types of producer cells is promising, as shown by various studies on different diseases. For instance, the macrophage-derived exosomes loaded with anti-cancer therapeutic compound PTX were used to treat multi-drug resistant cancer. PTX-loaded exosomes demonstrated an increased anti-cancer effect in murine lung tumor models^[25]^.

Small RNAs have been regarded as suitable regulators for modulating oncogene expression. However, the major drawback for their therapeutic use is the lack of an appropriate *in vivo* delivery system. Ohno *et al.*^[66]^ systemically injected let-7a miRNA-loaded exosomes into epidermal growth factor receptor (EGFR) - positive breast cancer xenograft mouse models. The exosome membranes were modified to express GE11 peptide for targeting EGFR; the engineered exosomes efficiently delivered miRNA and suppressed tumor phenotype^[66]^. Mendt *et al.*^[16]^ developed clinical grade siRNA-loaded MSC-derived exosomes called “iExosomes” which targeted KRAS (G12D) mutation in pancreatic cancer^[27]^. Additionally, iExosomes increased survival rate and reduced cancer phenotypes in various tumor models. Wang *et al.*^[121]^ showed that miR-185-enriched MSC-derived exosomes inhibit oral leukoplakia by reducing proliferation and angiogenesis in oral potentially malignant disorders.

Severe and chronic infections lead to inflammatory-related syndromes. Sepsis is associated with high mortality rate and has no standard therapeutic options. Choi *et al.*^[68] ^demonstrated that exosomes loaded with super-repressor IκB (srIκB), the constitutive active NF-κB signaling inhibition factor, using EXPLOR^®^ technology successfully inhibited pro-inflammatory responses *in vitro* and *in vivo* and prolonged the survival of septic animals. Inflammation is also a high-risk factor of preterm birth (PTB)^[122-124]^. Over the last 30 years, diverse therapeutic methods have been developed; however, only a few drugs related to obstetrics and gynecological diseases have reached clinical trials because of the difficulties in crossing the placental barrier^[125]^. By using cyclic recombinase-loaded exosomes, it was demonstrated that the engineered exosomes can pass through the placental barrier and efficiently deliver effective proteins to the fetus in the murine model^[20]^. These reports suggest that exosomes might be potent treatment agents for inflammation-induced PTB. Acute kidney injury is another immune-related disease with a high mortality rate and no definitive therapies^[126]^. Engineered exosomes delivering an anti-inflammatory modulator, interleukin-10, were shown to ameliorate kidney injury symptoms^[127]^.

One of the important reasons for exosomes to be considered as an attractive drug delivery vehicle is their ability to cross bio-barriers consisting of multiple cell layers^[128]^ as in PTB. Exosomes are rapidly emerging as a novel way to treat brain diseases as they can also cross the blood-brain barrier, one of the major hurdles for conventional drugs^[129,130]^. Alpha-synuclein (α-Syn) aggregation is the primary pathological phenotype of Parkinson`s disease. RVG-exosomes which specifically target the brain successfully delivered α-Syn-siRNAs to the brain of S129D α-Syn transgenic mice model**^[71]^**. Systemic administration of exosomes significantly reduced α-Syn protein aggregates in the brain of mice. In addition, catalase mRNA-loaded exosomes using EXOtic devices attenuated neurotoxicity and neuroinflammation in mouse brain.

## CONCLUSION

Three technologies are being used for producing therapeutic exosomes: naïve exosomes, delivering drugs directly into exosomes, and indirect loading of drugs into exosomes through genetic modification of producer cells. Several studies in academia and industry are using these techniques for developing therapeutic exosomes, and various cell lines are being investigated for optimal production of therapeutic exosomes.

The exosomes derived from stem cells including MSCs and NSCs have shown intrinsic therapeutic efficacy. Therefore, it is important to know the exact mechanisms of how these effects are derived into the exosomes from their parent cells. In addition, further studies related to scalability, consistent quality, and compatibility of the cells with cGMP guidelines should be performed before their clinical application.

With its ease of manipulation, HEK293 cell line is a key platform to produce therapeutic cargo-loaded exosomes along with improving the production process. However, the intrinsic effects of naïve HEK293-derived exosomes are still unclear; hence, in-depth studies are required to characterize HEK293-derived exosomes.

It has been shown that the molecular compositions of exosomes are dependent not only on the cell type but also on the physiological status of the producing cells. However, currently, it is not easy to distinguish the subtypes of exosomes. Furthermore, the exact *in vivo* distribution and stability of exosomes remain unknown, and additional in-depth studies with highly sensitive analytic methods are needed for a more accurate understanding of the mechanisms of action and to obtain more information on pharmacologically active components of exosomes^[102]^. In addition, engineering technologies should be further improved to enhance the activities and targeting ability of exosomes. Likewise, the human cells/cell lines used for biopharmaceutical productions should be free from safety and toxicity concerns including tumorigenicity and immunogenicity.

## DECLARATIONS

### Copyright

© The Author(s) 2021.

## References

[1] Théry C, Zitvogel L, Amigorena S (2002). Exosomes: composition, biogenesis and function. Nat Rev Immunol.

[2] Raposo G (2013). Stoorvogel W. Extracellular vesicles: exosomes, microvesicles, and friends. J Cell Biol.

[3] Tikhomirov R, Donnell BR, Catapano F (2020). Exosomes: from potential culprits to new therapeutic promise in the setting of cardiac fibrosis. Cells.

[4] Wiklander OPB, Brennan MÁ, Lötvall J, Breakefield XO, El Andaloussi S (2019). Advances in therapeutic applications of extracellular vesicles. Sci Transl Med.

[5] Babst M MVB vesicle formation: ESCRT-dependent, ESCRT-independent and everything in between. Curr Opin Cell Bio.

[6] Zhang Y, Liu Y, Liu H, Tang WH (2019). Exosomes: biogenesis, biologic function and clinical potential. Cell Biosci.

[7] Stenmark H (2009). Rab GTPases as coordinators of vesicle traffic. Nat Rev Mol Cell Biol.

[8] Sinha S, Hoshino D, Hong NH (2016). Cortactin promotes exosome secretion by controlling branched actin dynamics. J Cell Biol.

[9] Kalluri R, LeBleu VS (2020). The biology, function, and biomedical applications of exosomes. Science.

[10] Mayor S, Pagano RE (2007). Pathways of clathrin-independent endocytosis. Nat Rev Mol Cell Biol.

[11] Feng D, Zhao WL, Ye YY (2010). Cellular internalization of exosomes occurs through phagocytosis. Traffic.

[12] Svensson KJ, Christianson HC, Wittrup A (2013). Exosome uptake depends on ERK1/2-heat shock protein 27 signaling and lipid Raft-mediated endocytosis negatively regulated by caveolin-1. J Biol Chem.

[13] Tian T, Zhu YL, Zhou YY (2014). Exosome uptake through clathrin-mediated endocytosis and macropinocytosis and mediating miR-21 delivery. J Biol Chem.

[14] Parolini I, Federici C, Raggi C (2009). Microenvironmental pH is a key factor for exosome traffic in tumor cells. J Biol Chem.

[15] Delcayre A, Estelles A, Sperinde J (2005). Exosome display technology: applications to the development of new diagnostics and therapeutics. Blood Cells Mol Dis.

[16] Mendt M, Kamerkar S, Sugimoto H (2018). Generation and testing of clinical-grade exosomes for pancreatic cancer. JCI Insight.

[17] Zhu X, Badawi M, Pomeroy S (2017). Comprehensive toxicity and immunogenicity studies reveal minimal effects in mice following sustained dosing of extracellular vesicles derived from HEK293T cells. J Extracell Vesicles.

[18] Zhuang X, Xiang X, Grizzle W (2011). Treatment of brain inflammatory diseases by delivering exosome encapsulated anti-inflammatory drugs from the nasal region to the brain. Mol Ther.

[19] Alvarez-Erviti L, Seow Y, Yin H, Betts C, Lakhal S, Wood MJA (2011). Delivery of siRNA to the mouse brain by systemic injection of targeted exosomes. Nat Biotechnol.

[20] Sheller-Miller S, Choi K, Choi C, Menon R (2019). Cyclic-recombinase-reporter mouse model to determine exosome communication and function during pregnancy. Am J Obstet Gynecol.

[21] Song Y, Kim Y, Ha S (2020). The emerging role of exosomes as novel therapeutics: Biology, technologies, clinical applications, and the next. Am J Reprod Immunol.

[22] Lai RC, Arslan F, Tan SS (2010). Derivation and characterization of human fetal MSCs: an alternative cell source for large-scale production of cardioprotective microparticles. J Mol Cell Cardiol.

[23] Zhu L, Kalimuthu S, Gangadaran P (2017). Exosomes derived from natural killer cells exert therapeutic effect in melanoma. Theranostics.

[24] Sun D, Zhuang X, Xiang X (2010). A novel nanoparticle drug delivery system: the anti-inflammatory activity of curcumin is enhanced when encapsulated in exosomes. Mol Ther.

[25] Kim MS, Haney MJ, Zhao Y (2016). Development of exosome-encapsulated paclitaxel to overcome MDR in cancer cells. Nanomedicine.

[26] Lamichhane TN, Jeyaram A, Patel DB (2016). Oncogene knockdown via active loading of small RNAs into extracellular vesicles by sonication. Cell Mol Bioeng.

[27] Kamerkar S, LeBleu VS, Sugimoto H (2017). Exosomes facilitate therapeutic targeting of oncogenic KRAS in pancreatic cancer. Nature.

[28] Fuhrmann G, Serio A, Mazo M, Nair R, Stevens MM (2015). Active loading into extracellular vesicles significantly improves the cellular uptake and photodynamic effect of porphyrins. J Control Release.

[29] Hood JL (2016). Post isolation modification of exosomes for nanomedicine applications. Nanomedicine (Lond).

[30] Hood JL, Scott MJ, Wickline SA (2014). Maximizing exosome colloidal stability following electroporation. Anal Biochem.

[31] Haney MJ, Klyachko NL, Zhao Y (2015). Exosomes as drug delivery vehicles for Parkinson’s disease therapy. J Control Release.

[32] Luan X, Sansanaphongpricha K, Myers I, Chen H, Yuan H, Sun D (2017). Engineering exosomes as refined biological nanoplatforms for drug delivery. Acta Pharmacol Sin.

[33] Kojima R, Bojar D, Rizzi G (2018). Designer exosomes produced by implanted cells intracerebrally deliver therapeutic cargo for Parkinson’s disease treatment. Nat Commun.

[34] Wang Q, Yu J, Kadungure T, Beyene J, Zhang H, Lu Q (2018). ARMMs as a versatile platform for intracellular delivery of macromolecules. Nat Commun.

[35] Yim N, Ryu SW, Choi K (2016). Exosome engineering for efficient intracellular delivery of soluble proteins using optically reversible protein-protein interaction module. Nat Commun.

[36] Tian Y, Li S, Song J (2014). A doxorubicin delivery platform using engineered natural membrane vesicle exosomes for targeted tumor therapy. Biomaterials.

[37] Ibrahim AG, Cheng K, Marbán E (2014). Exosomes as critical agents of cardiac regeneration triggered by cell therapy. Stem Cell Reports.

[38] Ismail N, Wang Y, Dakhlallah D (2013). Macrophage microvesicles induce macrophage differentiation and miR-223 transfer. Blood.

[39] Taibi F, Metzinger-Le Meuth V, Massy ZA, Metzinger L (2014). miR-223: an inflammatory oncomiR enters the cardiovascular field. Biochim Biophys Acta.

[40] Wang X, Gu H, Qin D (2015). Exosomal miR-223 contributes to mesenchymal stem cell-elicited cardioprotection in polymicrobial sepsis. Sci Re.

[41] Xin H, Li Y, Cui Y, Yang JJ, Zhang ZG, Chopp M (2013). Systemic administration of exosomes released from mesenchymal stromal cells promote functional recovery and neurovascular plasticity after stroke in rats. J Cereb Blood Flow Metab.

[42] Feng Y, Huang W, Wani M, Yu X, Ashraf M (2014). Ischemic preconditioning potentiates the protective effect of stem cells through secretion of exosomes by targeting Mecp2 via miR-22. PLoS One.

[43] Liu L, Jin X, Hu CF, Li R, Zhou Z, Shen CX (2017). Exosomes derived from mesenchymal stem cells rescue myocardial ischaemia/reperfusion injury by inducing cardiomyocyte autophagy via AMPK and Akt pathways. Cell Physiol Biochem.

[44] Zhang Y, Chopp M, Meng Y (2015). Effect of exosomes derived from multipluripotent mesenchymal stromal cells on functional recovery and neurovascular plasticity in rats after traumatic brain injury. J Neurosurg.

[45] Kim DK, Nishida H, An SY, Shetty AK, Bartosh TJ, Prockop DJ (2016). Chromatographically isolated CD63 + CD81 + extracellular vesicles from mesenchymal stromal cells rescue cognitive impairments after TBI. Proc Natl Acad Sci U S A.

[46] Wang SS, Jia J, Wang Z (2018). Mesenchymal stem cell-derived extracellular vesicles suppresses iNOS expression and ameliorates neural impairment in Alzheimer’s disease mice. J Alzheimers Dis.

[47] Reza-Zaldivar EE, Hernandez-Sapiens MA, Gutierrez-Mercado YK (2019). Mesenchymal stem cell-derived exosomes promote neurogenesis and cognitive function recovery in a mouse model of Alzheimer’s disease. Neural Regen Res.

[48] Zhu YG, Feng XM, Abbott J (2014). Human mesenchymal stem cell microvesicles for treatment of Escherichia coli endotoxin-induced acute lung injury in mice. Stem Cells.

[49] Lai P, Chen X, Guo L (2018). A potent immunomodulatory role of exosomes derived from mesenchymal stromal cells in preventing cGVHD. J Hematol Oncol.

[50] He X, Dong Z, Cao Y (2019). MSC-derived exosome promotes M2 polarization and enhances vutaneous wound healing. Stem Cells Int.

[51] (2017). Nargesi A, Lerman LO, Eirin A. Mesenchymal stem cell-derived extracellular vesicles for kidney repair: current status and looming challenges. Stem. Cell Res Ther.

[52] Katakowski M, Buller B, Zheng X (2013). Exosomes from marrow stromal cells expressing miR-146b inhibit glioma growth. Cancer Lett.

[53] Melzer C, Rehn V, Yang Y, Bähre H, von der Ohe J, Hass R (2019). Taxol-loaded MSC-derived exosomes provide a therapeutic vehicle to target metastatic breast cancer and other carcinoma cells. Cancers (Basel).

[54] Xin H, Katakowski M, Wang F (2017). MicroRNA cluster miR-17-92 cluster in exosomes enhance neuroplasticity and functional recovery after stroke in rats. Stroke.

[55] Chen Z, Wang H, Xia Y, Yan F, Lu Y (2018). Therapeutic potential of mesenchymal cell-derived miRNA-150-5p-expressing exosomes in rheumatoid arthritis mediated by the modulation of MMP14 and VEGF. J Immunol.

[56] Yi X, Wei X, Lv H (2019). Exosomes derived from microRNA-30b-3p-overexpressing mesenchymal stem cells protect against lipopolysaccharide-induced acute lung injury by inhibiting SAA3. Exp Cell Res.

[57] Chen Y, Zhao Y, Chen W (2017). MicroRNA-133 overexpression promotes the therapeutic efficacy of mesenchymal stem cells on acute myocardial infarction. Stem Cell Res Ther.

[58] Rong Y, Liu W, Wang J (2019). Neural stem cell-derived small extracellular vesicles attenuate apoptosis and neuroinflammation after traumatic spinal cord injury by activating autophagy. Cell Death Dis.

[59] Webb RL, Kaiser EE, Jurgielewicz BJ (2018). Human neural stem cell extracellular vesicles improve recovery in a porcine model of ischemic stroke. Stroke.

[60] Li B, Liu J, Gu G, Han X, Zhang Q, Zhang W (2020). Impact of neural stem cell-derived extracellular vesicles on mitochondrial dysfunction, sirtuin 1 level, and synaptic deficits in Alzheimer’s disease. J Neurochem.

[61] Liu Q, Tan Y, Qu T, Zhang J (2020). Therapeutic mechanism of human neural stem cell-derived extracellular vesicles against hypoxia-reperfusion injury in vitro. Life Sci.

[62] Ma K, Xu H, Zhang J (2019). Insulin-like growth factor-1 enhances neuroprotective effects of neural stem cell exosomes after spinal cord injury via an miR-219a-2-3p/YY1 mechanism. Aging (Albany NY).

[63] Liu Y, Li D, Liu Z (2015). Targeted exosome-mediated delivery of opioid receptor Mu siRNA for the treatment of morphine relapse. Sci Rep.

[64] Kosaka N, Iguchi H, Yoshioka Y, Hagiwara K, Takeshita F, Ochiya T (2012). Competitive interactions of cancer cells and normal cells via secretory microRNAs. J Biol Chem.

[65] Mizrak A, Bolukbasi MF, Ozdener GB (2013). Genetically engineered microvesicles carrying suicide mRNA/protein inhibit schwannoma tumor growth. Mol Ther.

[66] Ohno S, Takanashi M, Sudo K (2013). Systemically injected exosomes targeted to EGFR deliver antitumor microRNA to breast cancer cells. Mol Ther.

[67] Faruqu FN, Xu L, Al-Jamal KT (2018). Preparation of exosomes for siRNA delivery to cancer cells. J Vis Exp.

[68] Choi H, Kim Y, Mirzaaghasi A (2020). Exosome-based delivery of super-repressor IkappaBalpha relieves sepsis-associated organ damage and mortality. Sci Adv.

[69] Kim H, Yun N, Mun D (2018). Cardiac-specific delivery by cardiac tissue-targeting peptide-expressing exosomes. Biochem Biophys Res Commun.

[70] Jahangard Y, Monfared H, Moradi A, Zare M, Mirnajafi-Zadeh J, Mowla SJ (2020). Therapeutic effects of transplanted exosomes containing miR-29b to a rat model of Alzheimer’s disease. Front Neurosci.

[71] Cooper JM, Wiklander PB, Nordin JZ (2014). Systemic exosomal siRNA delivery reduced alpha-synuclein aggregates in brains of transgenic mice. Mov Disord.

[72] Hsu DH, Paz P, Villaflor G Exosomes as a tumor vaccine: enhancing potency through direct loading of antigenic peptides. J Immunothe.

[73] Lu Z, Zuo B, Jing R (2017). Dendritic cell-derived exosomes elicit tumor regression in autochthonous hepatocellular carcinoma mouse models. J Hepatol.

[74] Gallet R, Dawkins J, Valle J (2017). Exosomes secreted by cardiosphere-derived cells reduce scarring, attenuate adverse remodelling, and improve function in acute and chronic porcine myocardial infarction. Eur Heart J.

[75] Aminzadeh MA, Rogers RG, Fournier M (2018). Exosome-mediated benefits of cell therapy in mouse and human models of duchenne muscular dystrophy. Stem Cell Reports.

[76] Zhang Q, Sun J, Huang Y (2019). Human amniotic epithelial cell-derived exosomes restore ovarian function by transferring microRNAs against apoptosis. Mol Ther Nucleic Acids.

[77] Zhao B, Zhang Y, Han S (2017). Exosomes derived from human amniotic epithelial cells accelerate wound healing and inhibit scar formation. J Mol Histol.

[78] Zhao B, Li X, Shi X (2018). Exosomal microRNAs derived from human amniotic epithelial cells accelerate wound healing by promoting the proliferation and migration of fibroblasts. Stem Cells Int.

[79] Tan JL, Lau SN, Leaw B (2018). Amnion epithelial cell-derived exosomes restrict lung injury and enhance endogenous lung repair. Stem Cells Transl Med.

[80] Royce SG, Patel KP, Mao W, Zhu D, Lim R, Samuel CS (2019). Serelaxin enhances the therapeutic effects of human amnion epithelial cell-derived exosomes in experimental models of lung disease. Br J Pharmacol.

[81] Fu W, Lei C, Liu S (2019). CAR exosomes derived from effector CAR-T cells have potent antitumour effects and low toxicity. Nat Commun.

[82] Yeo RW, Lai RC, Zhang B (2013). Mesenchymal stem cell: an efficient mass producer of exosomes for drug delivery. Adv Drug Deliv Rev.

[83] Can A, Celikkan FT, Cinar O (2017). Umbilical cord mesenchymal stromal cell transplantations: A systemic analysis of clinical trials. Cytotherapy.

[84] Lai RC, Arslan F, Lee MM (2010). Exosome secreted by MSC reduces myocardial ischemia/reperfusion injury. Stem Cell Res.

[85] Elgaz S, Kuçi Z, Kuçi S, Bönig H, Bader P (2019). Clinical use of mesenchymal stromal cells in the treatment of acute graft-versus-host disease. Transfus Med Hemother.

[86] Chakari-Khiavi F, Dolati S, Chakari-Khiavi A (2019). Prospects for the application of mesenchymal stem cells in Alzheimer’s disease treatment. Life Sci.

[87] Hu P, Yang Q, Wang Q (2019). Mesenchymal stromal cells-exosomes: a promising cell-free therapeutic tool for wound healing and cutaneous regeneration. Burns Trauma.

[88] Katsuda T, Ochiya T (2015). Molecular signatures of mesenchymal stem cell-derived extracellular vesicle-mediated tissue repair. Stem Cell Res Ther.

[89] Vogel A, Upadhya R, Shetty AK (2018). Neural stem cell derived extracellular vesicles: Attributes and prospects for treating neurodegenerative disorders. EBioMedicine.

[90] Baek G, Choi H, Kim Y, Lee HC, Choi C (2019). Mesenchymal stem cell-derived extracellular vesicles as therapeutics and as a drug delivery platform. Stem Cells Transl Med.

[91] Chen TS, Arslan F, Yin Y (2011). Enabling a robust scalable manufacturing process for therapeutic exosomes through oncogenic immortalization of human ESC-derived MSCs. J Transl Med.

[92] Cha JM, Shin EK, Sung JH (2018). Efficient scalable production of therapeutic microvesicles derived from human mesenchymal stem cells. Sci Rep.

[93] James S, Fox J, Afsari F

[94] Stevanato L, Corteling RL, Stroemer P (2009). c-MycERTAM transgene silencing in a genetically modified human neural stem cell line implanted into MCAo rodent brain. BMC Neurosci.

[95] Sinden JD, Hicks C, Stroemer P, Vishnubhatla I, Corteling R (2017). Human neural stem cell therapy for chronic ischemic stroke: charting progress from laboratory to patients. Stem Cells Dev.

[96] Serakinci N, Guldberg P, Burns JS (2004). Adult human mesenchymal stem cell as a target for neoplastic transformation. Oncogene.

[97] Bruno S, Collino F, Deregibus MC, Grange C, Tetta C, Camussi G (2013). Microvesicles derived from human bone marrow mesenchymal stem cells inhibit tumor growth. Stem Cells Dev.

[98] Roccaro AM, Sacco A, Maiso P (2013). BM mesenchymal stromal cell-derived exosomes facilitate multiple myeloma progression. J Clin Invest.

[99] Zhang X, Tu H, Yang Y, Fang L, Wu Q, Li J (2017). Mesenchymal stem cell-derived extracellular vesicles: roles in tumor growth, progression, and drug resistance. Stem Cells Int.

[100] Li J, Lee Y, Johansson HJ (2015). Serum-free culture alters the quantity and protein composition of neuroblastoma-derived extracellular vesicles. J Extracell Vesicles.

[101] Graham FL, Smiley J, Russell WC, Nairn R (1977). Characteristics of a human cell line transformed by DNA from human adenovirus type 5. J Gen Virol.

[102] Ferguson SW, Nguyen J (2016). Exosomes as therapeutics: The implications of molecular composition and exosomal heterogeneity. J Control Release.

[103] Vink T, Oudshoorn-Dickmann M, Roza M, Reitsma JJ, de Jong RN (2014). A simple, robust and highly efficient transient expression system for producing antibodies. Methods.

[104] Dumont J, Euwart D, Mei B, Estes S, Kshirsagar R (2016). Human cell lines for biopharmaceutical manufacturing: history, status, and future perspectives. Crit Rev Biotechnol.

[105] Li J, Chen X, Yi J (2016). Identification and characterization of 293T cell-derived exosomes by profiling the protein, mRNA and MicroRNA components. PLoS One.

[106] Ferguson S, Kim S, Lee C, Deci M, Nguyen J The phenotypic effects of exosomes secreted from distinct cellular sources: a comparative study based on miRNA composition. AAPS.

[107] Saleh AF, Lázaro-Ibáñez E, Forsgard MA (2019). Extracellular vesicles induce minimal hepatotoxicity and immunogenicity. Nanoscale.

[108] Chen S, Lv M, Fang S, Ye W, Gao Y, Xu Y (2018). Poly(I:C) enhanced anti-cervical cancer immunities induced by dendritic cells-derived exosomes. Int J Biol Macromol.

[109] Markov O, Oshchepkova A, Mironova N (2019). Immunotherapy based on dendritic cell-targeted/-derived extracellular vesicles-a novel strategy for enhancement of the anti-tumor immune response. Front Pharmacol.

[110] Pitt JM, André F, Amigorena S Dendritic cell-derived exosomes for cancer therapy. J Clin Inves.

[111] Gehrmann U, Hiltbrunner S, Georgoudaki AM, Karlsson MC, Näslund TI, Gabrielsson S Synergistic induction of adaptive antitumor immunity by codelivery of antigen with alpha-galactosylceramide on exosomes. Cancer Re.

[112] Wang L, Xie Y, Ahmed KA (2013). Exosomal pMHC-I complex targets T cell-based vaccine to directly stimulate CTL responses leading to antitumor immunity in transgenic FVBneuN and HLA-A2/HER2 mice and eradicating trastuzumab-resistant tumor in athymic nude mice. Breast Cancer Res Treat.

[113] Damo M, Wilson DS, Simeoni E, Hubbell JA (2015). TLR-3 stimulation improves anti-tumor immunity elicited by dendritic cell exosome-based vaccines in a murine model of melanoma. Sci Rep.

[114] Morse MA, Garst J, Osada T (2005). A phase I study of dexosome immunotherapy in patients with advanced non-small cell lung cancer. J Transl Med.

[115] Tian Y, Jiang X, Chen X, Shao Z, Yang W (2014). Doxorubicin-loaded magnetic silk fibroin nanoparticles for targeted therapy of multidrug-resistant cancer. Adv Mater.

[116] Escudier B, Dorval T, Chaput N (2005). Vaccination of metastatic melanoma patients with autologous dendritic cell (DC) derived-exosomes: results of thefirst phase I clinical trial. J Transl Med.

[117] Besse B, Charrier M, Lapierre V (2016). Dendritic cell-derived exosomes as maintenance immunotherapy after first line chemotherapy in NSCLC. Oncoimmunology.

[118] Gao W, Liu H, Yuan J (2016). Exosomes derived from mature dendritic cells increase endothelial inflammation and atherosclerosis via membrane TNF-alpha mediated NF-kappaB pathway. J Cell Mol Med.

[119] Ibrahim AGE, Li C, Rogers R (2019). Augmenting canonical Wnt signalling in therapeutically inert cells converts them into therapeutically potent exosome factories. Nat Biomed Eng.

[120] Fournier C, Martin F, Zitvogel L, Kroemer G, Galluzzi L, Apetoh L (2017). Trial watch: adoptively transferred cells for anticancer immunotherapy. Oncoimmunology.

[121] Wang L, Yin P, Wang J (2019). Delivery of mesenchymal stem cells-derived extracellular vesicles with enriched miR-185 inhibits progression of OPMD. Artif Cells Nanomed Biotechnol.

[122] Romero R, Espinoza J, Kusanovic JP (2006). The preterm parturition syndrome. BJOG.

[123] Romero R, Dey SK, Fisher SJ (2014). Preterm labor: one syndrome, many causes. Science.

[124] Xu Y, Romero R, Miller D (2018). Innate lymphoid cells at the human maternal-fetal interface in spontaneous preterm labor. Am J Reprod Immunol.

[125] Beck S, Wojdyla D, Say L (2010). The worldwide incidence of preterm birth: a systematic review of maternal mortality and morbidity. Bull World Health Organ.

[126] Ronco C, Bellomo R, Kellum JA (2019). Acute kidney injury. Lancet.

[127] Tang TT, Wang B, Wu M (2020). Extracellular vesicle-encapsulated IL-10 as novel nanotherapeutics against ischemic AKI. Sci Adv.

[128] Ha D, Yang N, Nadithe V (2016). Exosomes as therapeutic drug carriers and delivery vehicles across biological membranes: current perspectives and future challenges. Acta Pharm Sin B.

[129] Perets N, Hertz S, London M, Offen D (2018). Intranasal administration of exosomes derived from mesenchymal stem cells ameliorates autistic-like behaviors of BTBR mice. Mol Autism.

[130] Williams AM, Dennahy IS, Bhatti UF (2019). Mesenchymal stem cell-derived exosomes provide neuroprotection and improve long-term neurologic outcomes in a swine model of traumatic brain injury and hemorrhagic shock. J Neurotrauma.

